# Fatigue in Multiple Sclerosis: A Resting-State EEG Microstate Study

**DOI:** 10.1007/s10548-024-01053-3

**Published:** 2024-06-07

**Authors:** Sara Baldini, Arianna Sartori, Lucrezia Rossi, Anna Favero, Fulvio Pasquin, Alessandro Dinoto, Alessio Bratina, Antonio Bosco, Paolo Manganotti

**Affiliations:** 1 Department of Medicine, Surgery and Health Sciences, Neurology Unit, Cattinara University Hospital ASUGI, University of Trieste, Trieste, Italy; 2 Neurology Unit, Hospital of Gorizia, ASUGI, Gorizia, Italy; 3 Department of Neuroscience, Biomedicine and Movement Sciences, Neurology Unit, University of Verona, Verona, Italy

**Keywords:** Multiple Sclerosis, EEG Microstates, Frequency Bands, Fatigue

## Abstract

Fatigue affects approximately 80% of people with Multiple Sclerosis (PwMS) and can impact several domains of daily life. However, the neural underpinnings of fatigue in MS are still not completely clear. The aim of our study was to investigate the spontaneous large-scale networks functioning associated with fatigue in PwMS using the EEG microstate approach with a spectral decomposition. Forty-three relapsing–remitting MS patients and twenty-four healthy controls (HCs) were recruited. All participants underwent an administration of Modified Fatigue Impact scale (MFIS) and a 15-min resting-state high-density EEG recording. We compared the microstates of healthy subjects, fatigued (F-MS) and non-fatigued (nF-MS) patients with MS; correlations with clinical and behavioral fatigue scores were also analyzed. Microstates analysis showed six templates across groups and frequencies. We found that in the F-MS emerged a significant decrease of microstate F, associated to the salience network, in the broadband and in the beta band. Moreover, the microstate B, associated to the visual network, showed a significant increase in fatigued patients than healthy subjects in broadband and beta bands. The multiple linear regression showed that the high cognitive fatigue was predicted by both an increase and decrease, respectively, in delta band microstate B and beta band microstate F. On the other hand, higher physical fatigue was predicted with lower occurrence microstate F in beta band. The current findings suggest that in MS the higher level of fatigue might be related to a maladaptive functioning of the salience and visual network.

## Introduction

Fatigue, a feeling of lack of energy and tiredness not related to muscle weakness, is a common symptom in patients with Multiple Sclerosis (PwMS; Mathiowetz 2003; van der Vuurst de Vries et al. 2018). It can be present in up to 80% of patients with adult onset of MS and 30% in those with early age onset (Carroll et al. 2016), producing significant detrimental effects on the quality of life (Krupp 1989; Induruwa et al. 2012). MS fatigue also exacerbates other symptoms, is worsened by heat, and is chronic (Mathiowetz 2003). However, the neural correlates of fatigue symptoms in MS still remain poorly understood. Some studies have related fatigue in MS to structural damage of white matter (WM;Rocca et al. 2014; Bisecco et al. 2016) and grey matter (GM; Gobbi et al. 2014a, b; Calabrese and Castellaro 2017), brain atrophy (Calabrese et al. 2010) and functional brain connectivity changes (Bisecco et al. 2018; Jaeger et al. 2019; Stefancin et al. 2019; Wylie et al. 2020; Sobczak et al. 2022). In particular, resting-state functional connectivity (rsFC) investigations have found how PwMS that experiencing fatigue showed an increased connectivity in the posterior cingulate cortex (Finke et al. 2015), a reduction in the anterior cingulate cortex as well as between the caudate and somatosensory cortex (Finke et al. 2015; Jaeger et al. 2019). Fatigue severity has also correlated with increased functional connectivity of the basal ganglia to the medial prefrontal cortex, precuneus, and posterior cingulate cortex (Bisecco et al. 2018). Functional Magnetic Resonance Imaging (fMRI), a technique based on metabolic changes, was the main approach used to investigate the functional network organization in MS. However, the large-scale networks might be studied by other methods that, unlike the fMRI, directly measure neural activity. An advanced neurophysiological method for investigating the spatial and temporal properties of resting-state networks (RSNs) using a high-density EEG (hdEEG) is based on the concept of EEG microstates. Broadband spontaneous EEG activity at rest can be described by a limited number of scalp potential topographies (maps) that remain stable for a certain period of time (60–120 ms) before rapidly transitioning to a different topography that remains stable again. These discrete epochs of topographic stability have been referred to as *microstates*, suggesting that the scalp potential field reflects the momentary state of global neuronal activity, and that changes in the topography of this field indicate changes in the global coordination of neuronal activity over time (Michel and Koenig 2018). In a recent study, it has been demonstrated that microstate analysis decomposition within separate, narrow frequency bands could provide more fine-grained information for capturing the spatiotemporal complexity of multichannel EEG. This further analysis might be useful for identifying new neural mechanisms and possibly for biomarker discovery in neurological disease as MS. Moreover, recent works have revealed how PwMS showed a different microstates activation than healthy controls (Gschwind et al. 2016; Baldini et al. 2023), suggesting that this approach might be effective to investigate resting-state networks (RSNs) changes correlated to the disease and, more specifically, to particular symptoms such as fatigue. In this study, we employed a broadband and a spectral decomposition of EEG microstates to investigate the neural correlates of fatigue in PwMS and healthy controls (HCs). Differences between fatigued (F-MS) and non-fatigued MS patients (nF-MS) and correlations between temporal dynamic of microstates across frequency bands and fatigue scores were analyzed. We hypothesized that fatigued MS patients, with a low level of disability, show altered spontaneous fluctuation EEG topographies compared to non-fatigued MS patients.

## Materials and Methods

### Participants and Data Acquisition

Forty-three patients with relapsing–remitting multiple sclerosis (RRMS; revised McDonald Criteria Polman et al. 2011; Thompson et al. 2018) form with a low level of disability (EDSS < 3:mean ± SD;range: 1.1 ± 0.7;0–2) were selected among patients followed in our Multiple Sclerosis Centre (Clinical Unit of Neurology, Cattinara University Hospital ASUGI, Trieste Italy) from August 2020. The inclusion criteria were: age > 18 years, disease duration ≤ 10 years and without cognitive impairment assessed by the Brief International Cognitive Assessment for Multiple Sclerosis (BICAMS; Goretti et al. 2014; Langdon et al. 2012). Exclusion criteria were: a relapse or steroid treatment within the previous 30 days before the neuropsychological assessment and high-density EEG (hdEEG) recordings, cranial bone defects, history or signs of other neurological disorders (e.g., head injury, cerebrovascular disease, epilepsy, brain surgery, tumor, and major psychiatric diagnoses), and use of medications that could interfere with the neuropsychological evaluation. Twenty-four healthy subjects (HCs), matched for age and sex, were also enrolled. All participants underwent to high-density EEG (hdEEG) with 256-channel monopolar EGI Hydrocel Geodesic Sensor (Electrical Geodesics Inc., Eugene, United States) in resting-state condition, according the procedure in Baldini et al. (Baldini et al. 2023). The study was conducted according to the Declaration of Helsinki and was approved by local ethic committee.

### Fatigue Evaluation

Fatigue was assessed during the daytime in a quiet room with the Italian version of the Modified Fatigue Impact scale (MFIS;Kos et al. 2005). The MFIS (a self-administered 21 item questionnaire) is characterized by three sub-scores measuring the physical (MFIS-phys.; 9 questions, score [0, 36]), cognitive (MFIS-cogn.; 10 questions, score [0, 40]) and psychosocial (MFIS-psychosoc.; 2 questions, score [0, 8]) fatigue. The categorization of the patients in the fatigued vs non-fatigues was based on a recent work that has provided updated normative data (Strober et al. 2020). The authors have indicated specific cutoffs with respect to age, gender and level of education. This has permitted a more accurate identification of the PwMS with high level of fatigue. The neuropsychologist was blinded to clinical and radiological findings. All evaluations were performed before the hdEEG recordings.

### EEG Data Preprocessing

The offline preprocessing was performed as reported in Tomescu et al. (Tomescu et al. 2018). Briefly, the recordings were band-pass filtered between 1-40 Hz and the further analysis was conducted on 204 electrodes, excluding those on the check and nape. After the cardiac and oculomotor artifacts correction, the data were down-sampled to 125 Hz and then recomputed to common average-reference. For each subject, we selected 5 min of artifact-free EEG, excluding any epiloptogenic alterations. EEG data processing has performed by costumed MATLAB scripts (release 2012a, Mathworks Inc., Natick, MA). Each recording was, then, filtered into the 5 traditional EEG frequency bands: delta (1–4 Hz), theta (4–8 Hz), alpha1 (8–10 Hz), alpha2 (11–14) and beta (15–30 Hz). Filter design was as stated by Férat et al. (Férat et al. 2022); the only difference was for alpha band, in particular we analyzed:

- Alpha1 (8-10 Hz): Lower passband edge: 8.00; lower transition bandwidth: 2.00 Hz (12 dB cut-off frequency: 7.00 Hz); upper passband edge: 10.00 Hz; upper transition bandwidth: 3.00 Hz (12 dB cut-off frequency: 11.50 Hz);
- Alpha2 (11–14): Lower passband edge: 11.00; lower transition bandwidth: 2.00 Hz (12 dB cut-off frequency: 10.00 Hz); upper passband edge: 14.00 Hz; upper transition bandwidth: 3.00 Hz (12 dB cut-off frequency: 15.50 Hz).

### Microstates Analysis and Frequency Bands

**Fig. 1:**
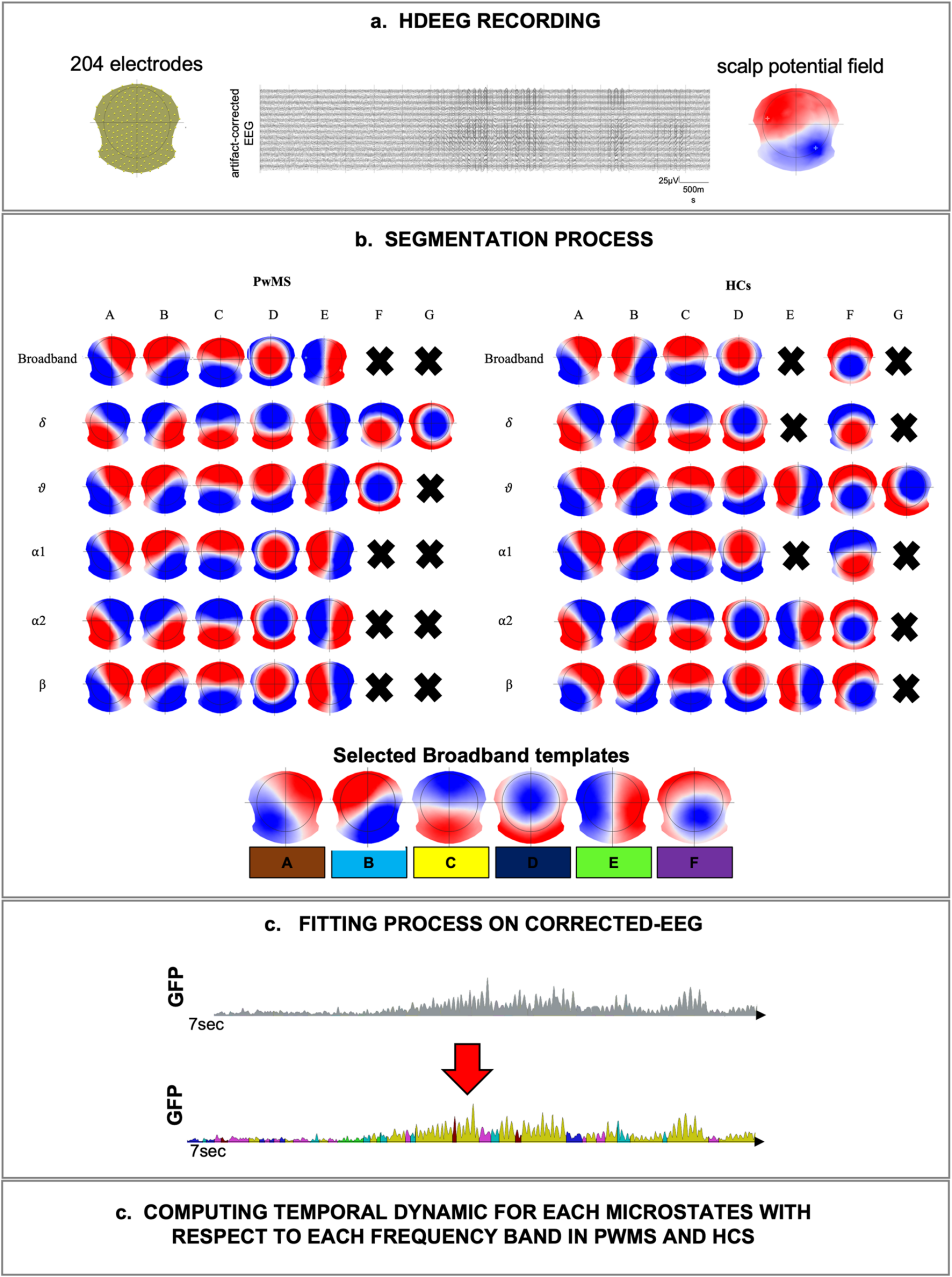
Schematic representation of the microstates analysis

The procedure of the microstates analysis has been performed in two steps: (1) the segmentation process, based on a k-mean cluster analysis, permitted to find the most dominant maps at individual and then at group level (across all subjects), and (2) the fitting process consisted into back-fitted the set of templates obtained at the group level on the whole artifact-free EEG of each individual in order to compute the temporal parameters of topographies (Tomescu et al. 2018). During the segmentation process, applied to each combination of frequency band (broadband, delta, theta, alpha1, alpha2, beta), maps with high spatial correlation are grouped together achieving the most representative topographical maps that best explain the variance in each cluster (Fig. 1a-b). The optimal number of clusters was obtained as reported in Tomescu et al. (2018). The most dominant templates were separately determined both for PwMS and HCs, identifying a set of microstates representing the EEG activity. Given the high spatial correlations between microstates across all frequencies and experimental groups, we selected for the following back-fitting process, a set of maps obtained on the broadband dataset and across all subjects in order to have a common reference. The fitting process was performed on the artifact-corrected EEG, separately for each analyzed frequency band (broadband, delta, theta, alpha1, alpha2, beta) (Fig. 1c-d). The individual participant’s EEG was labeled as a series of microstates and for each of them the following temporal parameters were computed; mean duration (MD), time coverage (TC), frequency of occurrence *per* minute (occ./min) and the global explained variance (GEV). The free academic software Cartool (release 3.70 [5292]) was used for the microstates analysis (Murray et al. 2008; Brunet et al. 2011).

### Statistical Analysis

We assessed whether temporal dynamic of microstates, obtained from the investigated frequency bands, show significant differences between fatigued (F-MS), non-fatigued MS patients (nF-MS) and healthy subjects (HCs). We performed the normality test (one-sample Kolmogorov–Smirnov test; α = 0.05) on the variable’s distributions taking in account all analyzed frequencies. Differences between microstate’s parameters of fatigued (n = 16), non-fatigued (n = 27) PwMS and healthy subjects (n = 24) were assed using Independent-Samples Kruskal–Wallis Test. It has been removed from this analysis three outliers with extreme values, one in fatigued and two in non-fatigued MS group. PwMS and HCs performances in MFIS were compared by Independent-Sample T-test. In order to assess how the clinical [age of disease onset, disease duration, EDSS and annualized relapse rate (ARR)] and fatigue data [MFIS-tot; MFIS-phys, MFIS-cogn.; MFIS-psychosoc.] were related to the microstates parameters [GEV; MD; TC; occ./min] in all investigated frequency bands (broadband, delta, theta, alpha1, alpha2, beta), Pearson correlations were performed separately for PwMS and HCs. Stepwise multiple linear regression models were then calculated (inclusion/exclusion probability levels for the stepwise procedure at < 0.05/ > 0.1). Multi-collinearity by means of the variance inflation factor (VIF) has been computed in order to estimate linear dependence between predictors. All statistical analyses run on SPSS (version 24.0, IBM Corporation, Aemonk, NY); alpha level = 0.05.

## Results

**Fig. 2:**
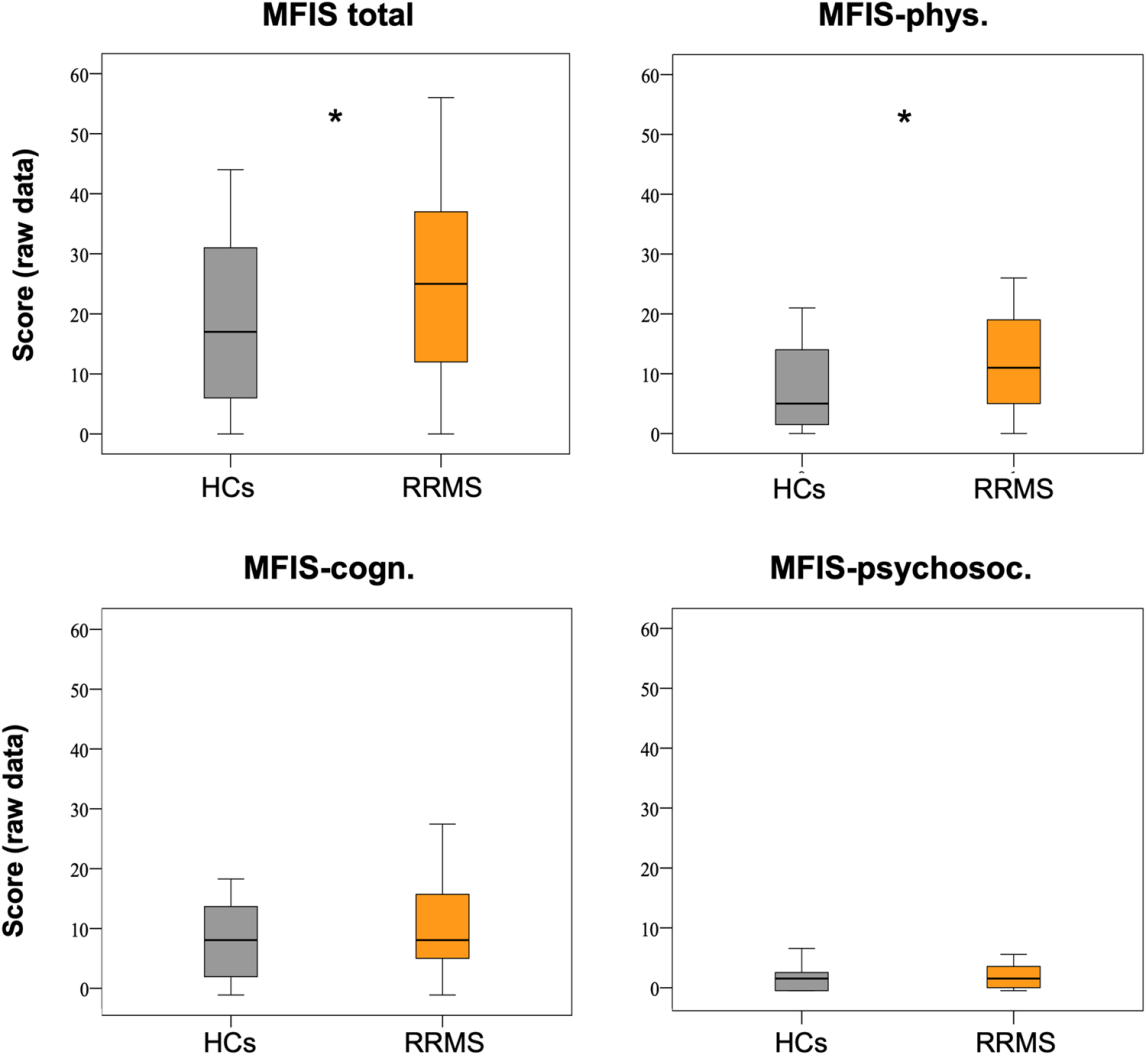
MFIS total and sub-scores in patients with MS and healthy controls. The boxplots show the MFIS total score, MFIS physical, cognitive and psychosocial fatigue in HCs (grey) and in PwMS (orange). The horizontal black lines in the middle denote median values; boxes extend from the 25th to the 75.^th^ percentile of each group's distribution of values; whiskers show the maximum and minimum values; * p < 0.05

We analyzed the data of 43 patients with RRMS and 24 matched HCs; detailed demographic, clinical and behavioral characteristics are reported in Table 1. Thirty-seven percent of patients showed high level of fatigue (16/43) as well as a 17% (4/24) in the control group; no difference was found between groups (χ^2^: p = 0.078; Table 2). The comparison of the MFIS-total scores distributions has shown significant higher values in the PwMS than HCs (Independent-Sample T-test: t = 1.753; df = 65; p = 0.042; Fig. 2). Taken in account the subscales of test, our clinical cohort reported a significant higher level of physical fatigue than what observed in control group (Independent-Sample T-test: t = 2.262; df = 65; p = 0.014; Fig. 2). The statistical analysis of the demographic features reported no differences between patient with or without high level of fatigue (Table 3).

**Table 1:** Summary of the demographic characteristics

Age (mean ± SD;range)		
RRMS	41 ± 11yrs;21–60	P = 0.797^#^
HCs	42 ± 12yrs;27–59	
Sex/Gender		
RRMS	22F/21 M	P = 0.343^$^
HCs	11F/13 M	
Education (mean ± SD;range)		
RRMS	14 ± 3yrs;8–22	P = 0.937^#^
HCs	21 ± 3yrs;18–25	
Disease Onset (mean ± SD;range)	34 ± 11yrs;16–59	
Disease Duration (mean ± SD;range)	7 ± 5yrs;0–19	
EDSS score at evaluation (mean ± SD;range)	1.1 ± 1;0–2	
ARR (median;range)	-0.034;-0.33–0.44	
Ongoing DMD (n; %)	37 (86%)	
Ist line	27/43 (63%)	
IInd line	10/43 (23%)	
No medication	6/43 (14%)	

*RRMS*: Relapse-Remitting Multiple Sclerosis; *HCs*: Healthy Controls; *EDSS*: Expanded Disability Status Scale; *ARR*: Annual Relapse Rate; *DMD*: Disease Modifying Drug; #: t-Test; $: chi-squared

**Table 2:** Fatigue assessment in PwMS and HCs

MFIS indexes	RRMS (mean ± SD;range)	HCs (mean ± SD;range)	p-value^#^	
MFIS-total	25 ± 16;0–56	19 ± 14;0–44	0.042	
MFIS-phys	12 ± 8;0–26	8 ± 7;0–21	0.014	
MFIS-cogn	11 ± 8;0–28	9 ± 7;0–19	0.145	
MFIS-psysoc	2 ± 2;0–6	2 ± 2;0–7	0.145	
MFIS classification^&^	RRMS (n;%)	HCs (n;%)	p-value^@^	
Fatigued	16;37%	4;17%	0.078	
Non-Fatigued	27;63%	20;83%		
BICAMS tests*	RRMS (mean ± SD;range)	nF-MS (mean ± SD;range)	F-MS (mean ± SD;range)	p-value^£^
SDMT	57 ± 8;38–81	58 ± 9;38–81	56 ± 8;43–67	0.450
CVLT-II	58 ± 11;40–84	56 ± 10;40–76	65 ± 11;41–84	0.014
BVMT-R	54 ± 8;38–76	53 ± 9;38–76	56 ± 5;50–64	0.138

*MFIS*: Modified Fatigue Impact scale; *MFIS-total*: total score of fatigue; *MFIS-phys*: score related to physical fatigue; *MFIS-cogn*: score related to cognitive fatigue; *MFIS-psysoc*: score related to psychosocial fatigue; *BICAMS*: Brief International Cognitive Assessment for Multiple Sclerosis; *SDMT*: Symbol Digit Modalities Test; *CVLT-II*: California Verbal Learning test II version; *BVMT-R*: Brief Visuospatial Memory Test-Revised; ^#^Indipendent-sample T-test; ^&^the classification of high level of fatigue, named *fatigued*, is based on the Sober et al. (2020); ^@^Chi-squared test; *the scores are expressed as standardized measures of T point; ^£^ nF-MS vs F-MS, Test U Mann–Whitney

**Table 3:** Demographic features of non-Fatigued (n = 27) and Fatigued (n = 16) patients with MS

Age (mean ± SD;range)		
nF-MS	40 ± 10yrs;21–60	P = 0.679^#^
F-MS	42 ± 13yrs;21–58	
Sex/Gender		
nF-MS	10F/14 M	P = 0.220^$^
F-MS	11F/5 M	
Education (mean ± SD;range)		
nF-MS	14 ± 4yrs;8–22	P = 0.988^#^
F-MS	14 ± 3yrs;8–19	
DO (mean ± SD;range)		
nF-MS	35 ± 12yrs;16–59	P = 0.489^#^
F-MS	38 ± 12yrs;19–55	
DD (mean ± SD;range)		
nF-MS	7 ± 5yrs;1–20	P = 0.376^#^
F-MS	5 ± 4yrs;2–18	
EDSS (mean ± SD;range)		
nF-MS	1.10 ± 0.7;0–2	P = 0.825^#^
F-MS	1.13 ± 0.8;0–2	
ARR (median;range)		
nF-MS	0.8 ± 1;0–3	P = 0.989^#^
F-MS	0.4 ± 0.3;0–4	
Ongoing DMD—nF-MS	Ongoing DMD—F-MS	
Ist line: 14/24 (58%)	Ist line: 10/16 (62%)	P = 0.354^$^
IInd line: 7/24 (29%)	IInd line: 3/16 (19%)	
No med.: 3/24 (13%)	No med: 3/16 (19%)	

*RRMS*: relapse-remitting multiple sclerosis; *HCs*: healthy controls; *DO*: Disease Onset; *DD*: Disease Duration; *EDSS*: Expanded Disability Status Scale; *ARR*: annual relapse rate; *DMD*: disease modifying drug; *no med*.: no medication; ^#^Test U Mann–Whitney; ^$^Chi-squared

### EEG Microstates and Fatigue in MS

**Fig. 3:**
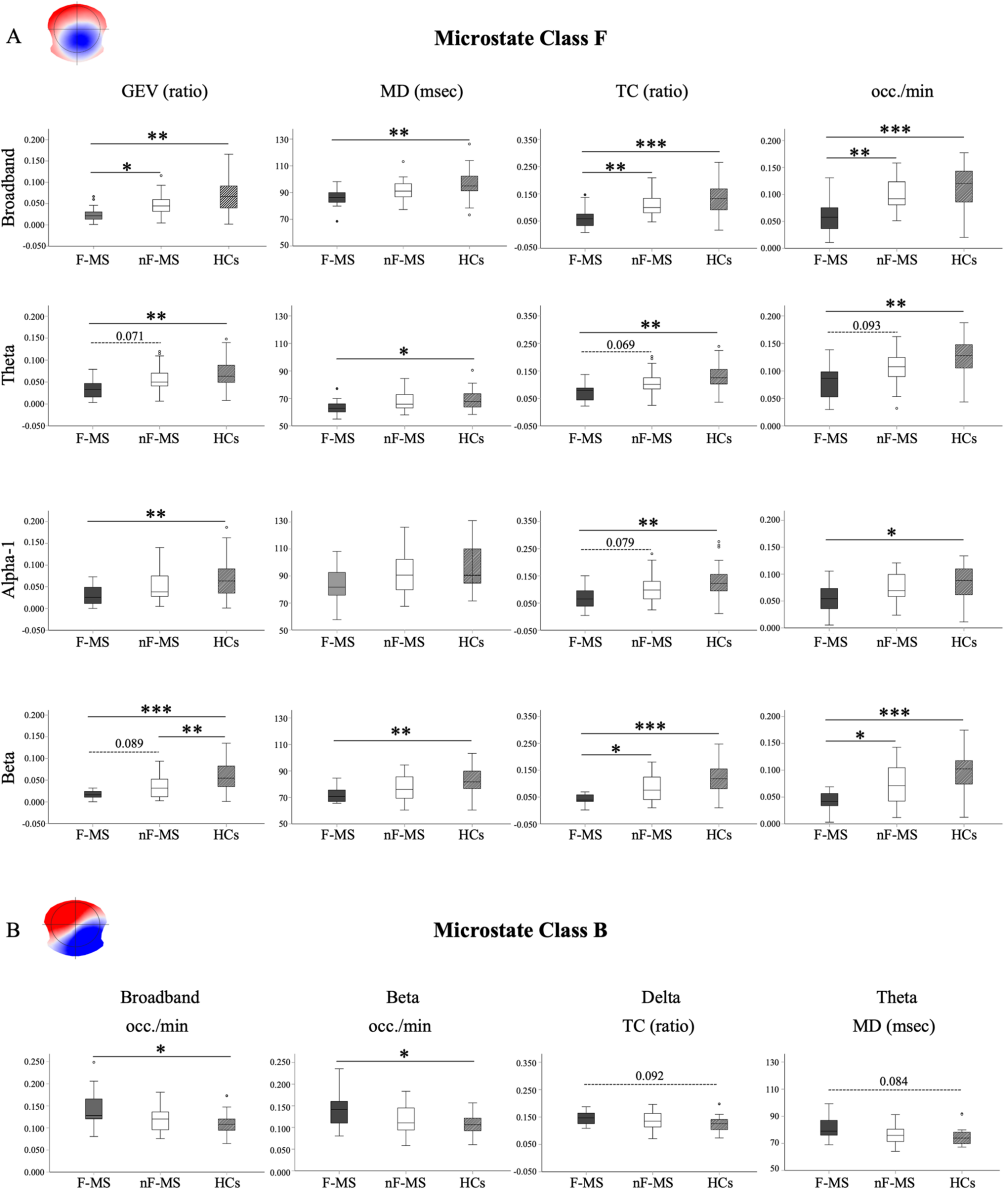
Temporal dynamic of the Microstate Class F and B in patients with high vs low level of fatigue and healthy controls**. A.** Differences in the temporal dynamic of the microstate F in fatigued, non-fatigued patients and healthy subjects, across frequency bands. **B.** The boxplots show differences in the microstate B across groups and frequency bands. The horizontal black lines in the middle denote median values; boxes extend from the 25th to the 75.^th^ percentile of each group's distribution of values; whiskers show the maximum and minimum values; * p < 0.05 **p < 0.01 *** < 0.001

Fatigued PwMS (F-MS: n = 15) showed a significant decrease in the temporal dynamic of microstate Class F compared to the those non-fatigued (nF-MS: n = 25) and healthy subjects (HCs; n = 24; Fig. 3). In particular, we found an alteration in the broadband (Independent-Samples Kruskal–Wallis Test; GEV p < 0.001; MD p = 0.02; TC p < 0.001; occ./min p < 0.001) and in beta band (Independent-Samples Kruskal–Wallis Test; GEV p < 0.001; MD p = 0.03; TC p < 0.001; occ./min p < 0.001). There were also statistical significances and marked trends in the theta (Independent-Samples Kruskal–Wallis Test; GEV p = 0.02; MD p = 0.032; TC p = 0.001; Occurrence/min p = 0.001) and alpha1 bands (Independent-Samples Kruskal–Wallis Test; GEV p = 0.008; TC p = 0.005; occ./min p = 0.013). We also observed a significant increase in fatigued patients than HCs of the microstate Class B in the broadband (Independent-Samples Kruskal–Wallis Test; occ./min p = 0.034) and beta band (Independent-Samples Kruskal–Wallis Test; occ./min p = 0.044). However, the differences between fatigued vs non fatigued did not reach any statistical significance. For the other maps, F-MS and nF-MS patients showed the same significant trends with respect to HCs that were observed in Baldini et al. (Baldini et al. 2023).

### Correlation Between Fatigue Levels and Microstates in PwMS and Healthy Controls

**Fig. 4:**
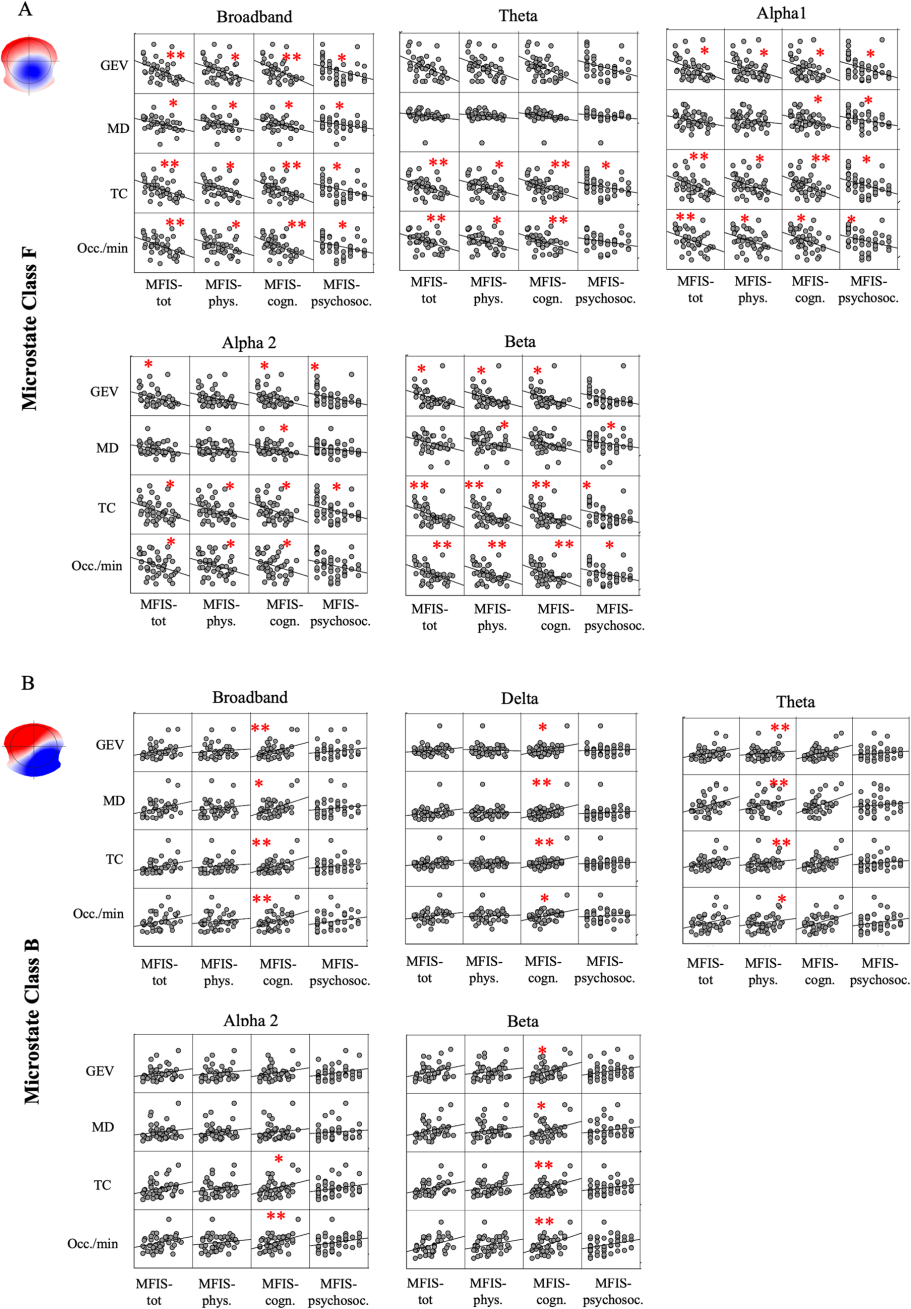
Correlations between microstates and MFIS scores in patients with MS. **A.** The correlation analysis between parameters of the microstate Class F with all MFIS scores, across frequency bands. **B.** The positive correlation between all parameters of the microstate Class B and cognitive fatigue across brain rhythms. MFIS-tot: MFIS total score; MFIS-Phys: MFIS score related to physical fatigue; MFIS-cogn: MFIS score related to cognitive fatigue; MFIS-Psysoc: MFIS score related to psychosocial fatigue; BB: broadband; GEV: Global Explained Variance; MD: Mean Duration; TC: Time Coverage; Occ./min: occurrence *per* minute

**Fig. 5:**
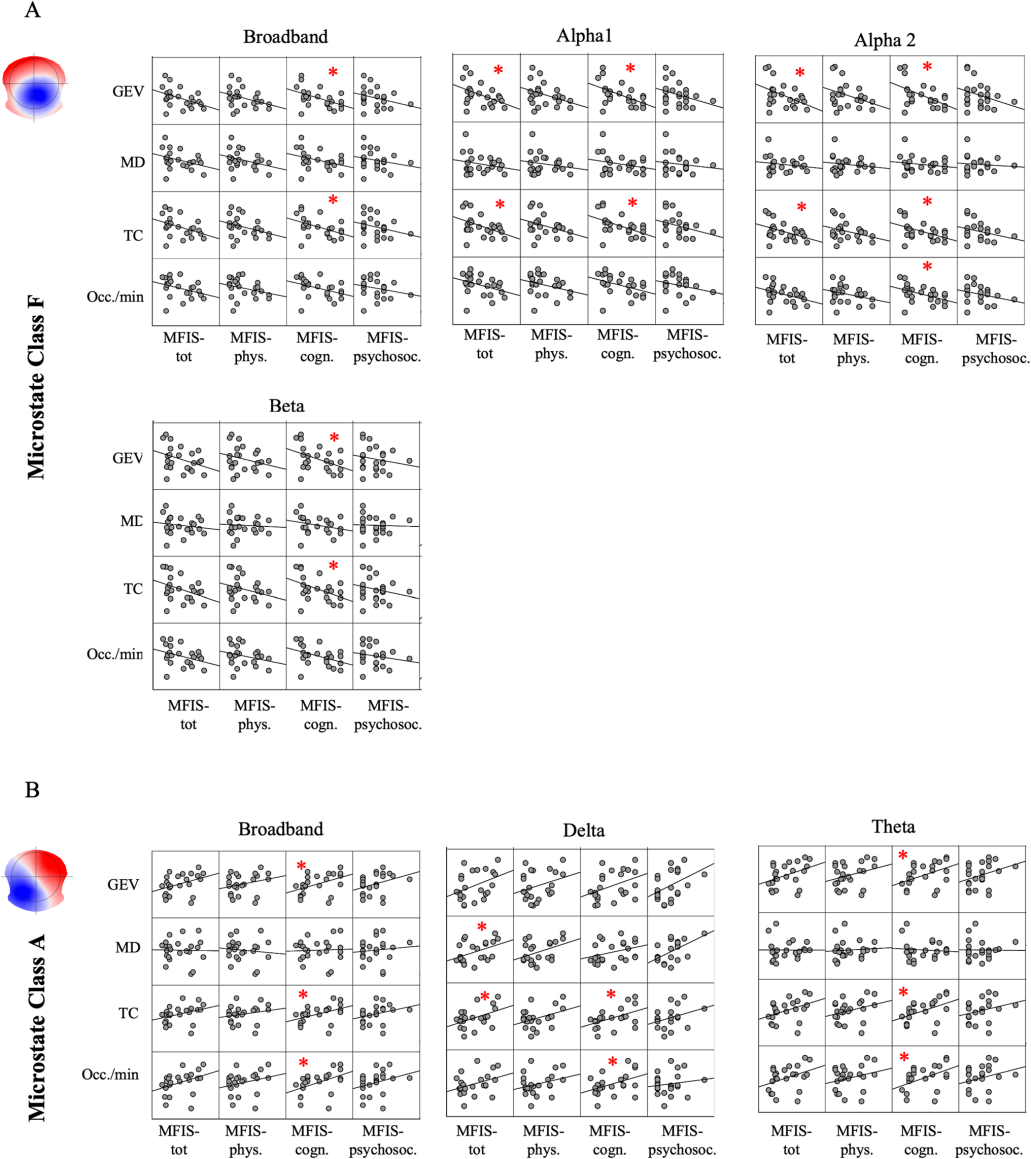
Correlations between microstates and MFIS cores in healthy controls. **A**. The negative correlation among the microstate F with mental fatigue in BB, alpha1 and 2 and beta bands. **B.** The positive correlation between several parameters of the microstate A and cognitive fatigue, across frequency bands. MFIS-tot: MFIS total score; MFIS-Phys: MFIS score related to physical fatigue; MFIS-cogn: MFIS score related to cognitive fatigue; MFIS-Psysoc: MFIS score related to psychosocial fatigue; BB: broadband; GEV: Global Explained Variance; MD: Mean Duration; TC: Time Coverage; Occ./min: occurrence *per* minute

We have also performed a correlation analysis between parameters of microstates and MFIS indexes, separately, for all PwMS (n = 43) and HCs (n = 24). For the patients, in the broadband, we found a significant negative and positive correlations, respectively, between Map-F and all MFIS indexes and Map-B only with cognitive fatigue (Fig. 4A-B). This pattern of association was observed, with some differences, across all frequency bands: a selective relationship concerning Map-B and the perception of mental tiredness (Fig. 4B) and a more spread involvement of Map-F among the different dimensions of fatigue (Fig. 4A). No differences were reported between clinical variables and MFIS indexes. In the healthy subjects, the correlation pattern was characterized by a significant correlation exclusively with cognitive fatigue, involving positively Map-A and negatively Map-F (Fig. 5A-B). This correlation was also found across frequency bands. Afterward, we set stepwise multiple linear regression models, respectively, for physical and cognitive fatigue as dependent variables across all patients with MS. We entered as potential predictors the parameters of the microstates mainly involved with these two types of fatigue as emerged from the correlation analysis. The strongest model we found predicted a high cognitive fatigue with a higher MD of Class B in delta band and a lower TC of Class F in beta band (P < 0.001; R^2^adj. = 30%, B = 0.360 for Map-B and B = -0.401 for Map-F VIF:1.039). On the other hand, higher physical fatigue was predicted with lower occurrence of Class F in beta band (P = 0.018; R^2^adj. = 15%, B = -0.414 VIF:1.000).

## Discussion

In the present study, we have investigated how the level of fatigue in patients with RRMS might have a neurophysiological correlate with the spontaneous fluctuations of neuronal activity in large-scale networks. Our results essentially showed, for a general perception of fatigue, a major involvement of the microstate Class F (salience network; SN) with a secondary contribution of the microstate Class B (visual network). We hypothesized that a lower activation of the salience network (Map-F) may play a role to induce a detrimental feeling of fatigue, both physically and cognitively. On the other hand, an increased presence of the Map-B could subserve the mental weariness, suggesting a possible type-dependent modulation of fatigue that it was further supported by the correlation results. These different microstates activations in respect to MFIS scores might be the expression of a maladaptive response of neuronal networks that, in the end, lead to a marked feeling of lack of energy and tiredness. Interestingly, in the healthy subjects was also observed that a lower activation of the Map-F and a higher activation of the Map-A were associated to higher level of cognitive fatigue. Together, these findings might suggest a possible shared pathway between PwMS and HCs with a less activation of the Map-F when the level of fatigue increases, regardless to the type of fatigue, and a discriminative feature with respect to the mental tiredness. Indeed, we have observed a distinct activation of the two sensory microstates: Map-A (auditory network) for healthy subjects and Map-B for patients with MS.

The microstate Class F has been associated to a strong activation in the dorsal anterior cingulate cortex (ACC, BA32) extending to the superior frontal gyrus with also a bilateral detection in the middle frontal gyrus and insula as reported in Custo et al. (Custo et al. 2017). Some of these brain areas were found to be involved in the cognitive fatigue. In literature, it has been identified that several brain regions appear to be related to cognitive fatigue, leading to hypothesize a “fatigue network”. This neuronal circuitry includes the striatum of the basal ganglia, the dorsolateral prefrontal cortex (DLPFC), the dorsal anterior cingulate cortex (dACC), the ventro-medial prefrontal cortex (vmPFC) and the anterior insula (Wylie et al. 2017; Bisecco et al. 2018; Müller and Apps 2019; Stefancin et al. 2019). These areas are functionally connected and their connectivity changes as a function of cognitive fatigue. Interestingly, Wylei et al.(Wylie et al. 2020) have induced mental fatigue in healthy subject and then applied a task-fMRI paradigm, finding that dACC and insula showed a decreased connectivity with frontal areas but an increased connectivity with inferior parietal lobule as function of cognitive fatigue (Wylie et al. 2020). The SN is interconnected with the fronto-parietal network, which involves intraparietal sulcus and superior parietal lobe and mediates goal-directed, suggesting a possible deficit of the top–down attention in the fatigued PwMS. Interestingly, the fronto-parital network is a main target of the therapeutic strategies to reduce efficaciously fatigue in MS (Ferrucci et al. 2014; Tecchio et al. 2014; Cancelli et al. 2018). In a recent rsFC study, it has been also found differences in posterior SN in fatigued MS patients, suggesting how the higher level of fatigue might be related to deficits in interoceptive and viscero-autonomic awareness (Sobczak et al. 2022). Moreover, the SN is involved in the neural process behind switching between DMN and the central executive network (CEN; Sridharan et al. 2008; Menon and Uddin 2010; Goulden et al. 2014) that is critical to segregate the most relevant internal and extrapersonal stimuli in order to guide behavior (Menon and Uddin 2010). From a metacognitive viewpoint, an altered salience network functioning might thus induce a possible mismatch between expected and actual stimuli then lead to the subjective experience of detrimental fatigue so often seen in MS patients (Leocani et al. 2008; Manjaly et al. 2019). This view might be further supported by the evidence that during a high cognitive load condition task, PwMS did not show more activation in superior and middle frontal gyri, insula, and superior temporal gyrus but, on the contrary, the MS group showed continued activation of more posterior regions (i.e., precuneus, lingual gyrus, and middle occipital gyrus) with no improvement in the task and more fatigue, compared to the healthy subjects (Chen et al. 2020). Our results are consistent with these observations, supporting the hypothesis that a lower presence of Map-F could represent changes in the spontaneous activations of the SN that might not permit to use more efficiently the cerebral resources, affected by the disease, and trigger a perception of lack of energy in MS. Noteworthy, an involvement of the Map-F was also found in the healthy subjects, at least for the mental weariness. However, the involvement was lesser marked and most probably temporary than what observed in the MS since the fatigue is one of the main symptoms.

In our study, we also found a significant increased activation of the Map-B, mainly related with the occipital areas, in fatigued MS patients than healthy individuals that, with Map-F, has shown a predictive role for the cognitive fatigue. In literature, only Gschwind et al. (2016) have correlated indexes of fatigue (FSMC: Fatigue Scale of Motor and Cognitive) with spontaneous fluctuation of microstates, finding a similar trend with a significant association between cognitive fatigue and Map-B in RRMS patients (Gschwind et al. 2016). Moreover, the microstate Class B has been related to certain cognitive abilities (e.g. fluid intelligence), self-visualization, autobiographical memory and scene visualization, supporting a possible link of this large-scale network with non-physical fatigue. However, these brain functions most probably are mediated by an interplay with other microstates mainly associated to precunes, posterior cingulate cortex, angular gyrus (Santarnecchi et al. 2017; Bréchet et al. 2020; Tarailis et al. 2023). According with these studies, we might postulate that a spontaneous decrease in the temporal dynamic of the Map-F together with a higher activation of Map-B may be an electrophysiological signature of maladaptive neuronal reorganization subserving detrimental level of both physical and cognitive fatigue in MS. Another crucial RSN related to the fatigue in MS is the DMN. Some studies have found in fatigued MS patients altered rsFC in the DMN, in particular both an increased rsFC in the PCC and a reduced rsFC in the ACC compared to nF-MS patients (Finke et al. 2015; Bisecco et al. 2018; Jaeger et al. 2019; Stefancin et al. 2019). In contrast, our study did not reveal significant fluctuations in the microstates associated to the DMN (Map-C and E) than fatigue levels. A possible explanation could be due to the different methodological approaches (high temporal vs high spatial resolution, resting-state vs task condition), assessment of fatigue (MFIS vs FSS) and the clinical cohort studied (RRMS/SPMS).

To capture fine details of the spatiotemporal complex multichannel EEG analysis, we have also applied a spectral decomposition of the EEG microstates. The comparison has shown that the main differences for the microstate Class F and Class B, observed in the fatigued-MS patients, emerged in the broadband and beta rhythms. Noteworthy, the correlation analysis has reported other contributions from different frequency bands. The negative association between the Map-F and the physical tiredness was also evident in the alpha-1 and with less marked significances in the theta and alpha-2 rhythms. Moreover, a clear participation of the frequency alpha-1 was observed both for the cognitive and psychosocial fatigue. On the other hand, the positive correlation of the Map-B with the cognitive fatigue was appreciated in the delta and theta bands. In summary, the microstate F did not show selective association to the different dimensions of fatigue investigated with MFIS, reporting particular strength in beta and alpha-1 bands, beyond the broadband. Instead, for the cognitive fatigue there is also the contribution of the Map-B especially in the low frequency bands. These data were supported by the multiple linear regression analysis that showed a strong prediction of the cognitive fatigue by a higher mean duration of microstate B in delta band and a lower time coverage of microstate F in beta band. At the same time, for the physical fatigue, the lower occurrence of microstate F in beta band was the main predictor. Medium/High (alpha-1/beta) frequencies are the prominent spontaneous oscillations in the insula as well as for ACC and superior/middle frontal gyrus (Capilla et al. 2022), supporting one more a critical role of the SN in the feeling of fatigue in MS. Beta oscillations have been hypothesized to underlie directed causal information flow from one brain region to another and may contribute to transitioning latent neuronal ensembles into “active” representations (Spitzer and Haegens 2017) as well as the subsequent maintenance of information in cell assemblies (Engel and Fries 2010). Moreover, a functional connectivity study in the resting state with eyes open has observed changes in tandem with fatigue symptoms in the beta band (Vecchio et al. 2017). The authors found that with increasing fatigue, the left parietal-occipital-temporal brain network showed a more evident impairment of integration than segregation especially in the beta-mediated sensorimotor communication (Vecchio et al. 2017). On the other side, alpha rhythm reflects the state of relaxation and wakefulness that is blocked during focusing attention, external stimulation or visual input (Putilov and Donskaya 2014). It is widely accepted that alpha rhythm intensifies as the brain transformed from normal into fatigue (Lal and Craig 2002; Craig et al. 2012) and it is considered to be the most sensitive indicator of brain fatigue (Li et al. 2020; Linnhoff et al. 2023). Besides, the brain areas associated to the Map-B, located in the occipital lobe that is demonstrated to be involved in the MS fatigue (Gobbi et al. 2014b), usually show both a spontaneous and dominant oscillation in the alpha rhythm. Our results, instead, reported a main contribution of the Map-B in beta and lower frequency bands and this pattern might suggest a possible alteration in the allocation of sensory attention (Jensen and Mazaheri 2010), contributing to the perception of mental fatigue.

This study has some limitations. Firstly, concepts of fatigue vary remarkably in the literature, leading to heterogeneous concepts with different operationalized definitions and, therefore, with various fatigue questionaries in MS. In our study, we have used a subjective perception of fatigue that requires a cognitive perspective, in particular, concepts of enteroception and metacognition (Manjaly et al. 2019). Secondary, the EEG has low power to localize deep structures as basal ganglia and thalamus that are found to play a critical role in the MS-related fatigue (Bisecco et al. 2018; Jaeger et al. 2019; Stefancin et al. 2019). We did not rule out a possible effect of these subcortical structures, taking in account that in the SN visceromotor inputs informative of the current body state reach the fronto-insular cortex via vagus nerve, autonomic afferent nuclei, thalamus, the dorsal posterior insular cortex and mid-insula (Uddin 2015). Lastly, the small number of treatment-related groups may be a limitation, as we could not accurately exclude possible effects on microstates variables. Nevertheless, taking in account the different type of drugs, it might be difficult to detect any influences. Our study provides new insights to the resting-state networks functioning with respect to level of fatigue in multiple sclerosis. Specific patterns in the temporal dynamic of microstates might anticipate some clinical manifestations as fatigue in PwMS and could be interesting to investigate their possible changes in the activation as function of treatment’s protocol with transcranial direct current (tDCS). A higher level of fatigue was related to changes in the salience network functioning and a recruitment of more posterior/visual areas possibly subserved the cognitive fatigue. These results might help to improve the understanding of fatigue-related pathomechanisms in MS, stimulating new perspectives of the treatments options.
